# A Tool for Rating Chronic Disease Prevention and Public Health Interventions

**DOI:** 10.5888/pcd10.130173

**Published:** 2013-12-12

**Authors:** Cheryl Kelly, Darcell Scharff, Jessi LaRose, Nikole Lobb Dougherty, Amy Stringer Hessel, Ross C. Brownson

**Affiliations:** Author Affiliations: Darcell Scharff, Saint Louis University, St. Louis, Missouri; Jessi LaRose, Amy Stringer Hessel, Missouri Foundation for Health, St. Louis, Missouri; Nikole Lobb Dougherty, Ross C. Brownson, Washington University in St. Louis, St. Louis, Missouri.

## Abstract

Bridging the gap between research and practice requires more than evaluating the effectiveness of interventions in controlled studies. To bridge this gap, evidence needs to be defined in different ways, and opportunities need to be provided for practice-based evidence to be replicated and disseminated. Community-based interventions are often not conducted or evaluated in controlled settings, yet they provide more real-world context and have the potential to have a greater effect on population health than findings from controlled studies that are limited in generalizability. The purpose of this article is to describe an approach to identify community-based programs and interventions that have the potential for replication and dissemination. In our study, such interventions met criteria in 3 primary domains: innovativeness, effectiveness, and sustainability. The criteria and tool developed were applied to 2 obesity-prevention programs to demonstrate the usefulness of the tool for identifying potential programs for replication and dissemination, contributing to practice-based evidence. Funders, practitioners, and researchers can apply these criteria to identify programs, environmental changes, or policies that may be replicated and disseminated.

## Introduction

The gap between research and practice in public health has been well documented (1). With calls from the Institute of Medicine, National Institutes of Health, and Centers for Disease Control and Prevention (CDC), public health institutions are attempting to accelerate the path from intervention development and evaluation to dissemination and adoption by practitioners. It has been suggested that it takes 17 years for 14% of original clinical research to reach patients (2). Researchers are pushing the scientific field to redefine evidence in a way that will expedite the process.

Historically, public health evidence has been developed on the basis of studies that meet rigorous research standards (eg, experimental designs) (3). Although these studies are important for determining efficacy or effectiveness (internal validity), they often lack consideration of external validity or generalizability and applicability outside of a controlled setting (due to unrepresentative samples and lack of consideration for real-world contextual variables) (4–9). Evaluations of community-based programs that lack internal validity (ie, no control groups) but still have a large effect on a population are often excluded from evidence-based research. Publications of rigorous, less generalizable findings often trump publication of practice-based research that can speed up dissemination so that behavior change can be made.

There is a need for greater recognition of “practice-based evidence” where knowledge is derived from real-world settings often via evaluation of natural experiments. This type of evidence addresses the lack of generalizability issue resulting from methodologically strong research designs by producing results that are relevant and applicable to the community (7).

Recognizing the need to ensure that more practice-based evidence is recognized as valid and disseminated appropriately, researchers and practitioners have suggested criteria for identifying effective, promising, and emerging intervention strategies ready for dissemination and replication (3,10,11). For example, Glasgow and colleagues developed evaluation criteria that state that a program is ready for dissemination and replication when it has the potential to reach a large audience; can be broadly adopted and implemented by different populations, settings, or both; and engages the community in the process (7,9,10,12). In the usual published literature, many elements of external validity are missing (13,14), which indicates a call for new tools and approaches for increasing the rate of disseminating promising practices.

Another example is the use of Evaluability Assessments, which have recently reemerged as a way to guide investments in evaluation and research by identifying interventions that are ready for further evaluation and research (15). The Robert Wood Johnson Foundation and several partners have used Evaluability Assessments in the area of childhood obesity. To be considered for further evaluation, projects must meet several criteria, including demonstrating potential for impact, plausibility to reach a large portion of the population, and feasibility and transportability (15). The process identified 20 obesity-related projects over a 2-year period that were promising but represented only 4.4% of the total projects examined. These projects are being implemented and evaluated using rigorous evaluation and research methods.

Others have devised an evidence typology highlighting interventions that have the potential for public health impact but would have otherwise gone unnoticed (3,10,11,14). For example, the Center of Excellence for Training and Research Translation (Center TRT) developed criteria for reviewing and categorizing interventions. Center TRT uses a systematic process to “identify, review, translate, and disseminate interventions that are supported by evidence of potential for public health impact” (12). Intervention program developers submit published data or evaluation reports for review, and Center TRT and an expert panel categorize interventions as research-tested interventions, practice-based interventions, emerging interventions, or not ready for dissemination. Center TRT’s criteria allow unpublished evaluation results to be considered. 

Lastly, assessing project sustainability is key to identifying programs and interventions ready for replication and dissemination. Sustainability is defined as “maintaining programming and its benefits over time” (16). Positive outcomes of community-based interventions can be achieved only if effective programs, partnerships, and organizational practices are sustained over time (16–18). Funders are increasingly concerned with whether the public health interventions they support have the potential for intended benefits to extend beyond funding periods. Many funders (eg, CDC, Robert Wood Johnson Foundation) often implement tiered funding approaches for multiyear grants to ensure that grantees seek additional financial support and require that interventions demonstrate that program components will be sustained.

Schell and colleagues identified core domains of a conceptual framework for public health program capacity for sustainability (16). These core domains were political support, funding stability, partnerships, organizational capacity, program evaluation, program adaptation, communications, public health impacts, and strategic planning (18). Collectively, these criteria are a resource to help foundations and practitioners identify programs and interventions that may be ready for replication and dissemination. However, foundations and practitioners need a tool or approach for applying these criteria to local programs and rating the degree to which programs meet these criteria. Often, programs are funded with the intent to reach a particular population, improve health outcomes, and sustain programs. As programs show potential for achieving these goals, foundations and practitioners want to disseminate and replicate programs in similar communities.

## An Approach for Selecting Programs to Be Replicated and Disseminated

The Missouri Foundation for Health (MFH) established the Model Practice Building (MPB) initiative in 2007 to support previously funded healthy eating and physical activity programs that showed potential for becoming sustainable, long-term programs. The MPB initiative grew from the Institute of Medicine’s recommendation that foundations help build the field of obesity prevention by 1) monitoring program progress, 2) evaluating programs and policies, 3) disseminating promising practices, and 4) providing leadership and sustained commitment to obesity prevention (19).

The MPB initiative provided a mechanism for growing local solutions that could have statewide or national significance. Nineteen organizations were funded for 3 years. A goal of this funding was to identify projects that could be classified as model practices using criteria from the field and could be disseminated and replicated across the state. To meet this goal, an external university-based evaluation and dissemination team developed an approach to select interventions that MFH could call model practices.

On the basis of the literature, criteria for selecting model practice interventions were reviewed and categorized into 3 domains: innovativeness, effectiveness, and sustainability. Table 1 lists the criteria in each domain and the previous research on which criteria were based. A project was considered innovative to the extent that it met 6 criteria (eg, implemented activities with a new population, implemented an environmental or policy intervention). A project was considered effective to the extent that it met 5 criteria (eg, project’s ability to reach target population, project’s ability to measure project effectiveness). Sustainable projects had evidence of political support, funding stability, community partnerships, organizational capacity, program evaluation, program adaptation, communication, and strategic planning. The stronger the evidence for the criteria in each of these domains, the more likely programs were to represent a good investment suitable for dissemination and replication (Table 1).

**Table 1:** Model Practice Criteria Applied to 2 Obesity-Prevention Programs

Criterion	Criterion Adapted From (Reference No.)	PCHC	Trailnet HAVC Initiative

		Score (%)^a^	Score (%)^a^
**Innovativeness^b^**			
1. Environmental or policy approach	Brennan et al (11), Leviton et al (15)	3 (100)	3 (100)
2. Activities with a new population	Green and Glasgow (7)	1 (33)	1 (33)
3. Activities in a new setting	Green and Glasgow (7), Klesges et al (13)	1 (33)	2 (67)
4. Address populations with health disparities	Brennan et al (11)	3 (100)	2 (67)
5. Engage partners from diverse sectors	Green and Glasgow (7)	3 (100)	3 (100)
6. Gathered evidence to guide the adaptation of an existing program or policy for a specific community	Klesges et al (13)	3 (100)	3 (100)
**Total**	**NA**	**14 (78)**	**14 (78)**
**Effectiveness^b^**			
1. Ability to reach the target population	Green and Glasgow (7), Klesges et al (13), Hoehner et al (14)	2 (67)	2 (67)
2. Design linked to an existing evidence-based intervention or theory	Brennan et al (11), Klesges et al (13)	3 (100)	3 (100)
3. Fidelity of the program to evidence-based interventions	Brennan et al (11), Klesges et al (13)	1 (33)	2 (67)
4. Program can demonstrate that it has processes and procedures in place to measure project effectiveness	Hoehner et al (14)	2 (67)	2 (67)
5. Degree to which the program’s effectiveness is demonstrated by its own internal evaluation results	Brennan et al (11), Hoehner et al (14)	1 (33)	2 (67)
**Total**	**NA**	**9 (60)**	**11 (74)**
**Sustainability^c^**			
1. Political support	Shediac-Rizkallah and Bone (18)	5.7 (81)	5.5 (79)
2. Funding stability	Shediac-Rizkallah and Bone (18)	4.9 (70)	2.7 (39)
3. Community partnerships	Shediac-Rizkallah and Bone (18)	5.6 (80)	5.7 (81)
4. Organizational capacity	Shediac-Rizkallah and Bone (18)	6.3 (90)	6.4 (91)
5. Program evaluation	Shediac-Rizkallah and Bone (18)	5.9 (84)	5.7 (81)
6. Program adaptation	Shediac-Rizkallah and Bone (18)	5.7 (81)	6.5 (93)
7. Communication	Shediac-Rizkallah and Bone (18)	6.0 (86)	5.7 (81)
8. Strategic planning	Shediac-Rizkallah and Bone (18)	5.2 (74)	5.1 (73)
**Total**	**NA**	**45.3 (81)**	**43.3 (77)**
**Average Model Practice Building Score**	**NA**	**73**	**76**

Abbreviation: PCHC, Polk County Health Center; HAVC Initiative, Healthy, Active, and Vibrant Communities Initiative; NA, not applicable.

a Percentage is calculated by dividing the score by the total points possible (equalize the scores).

b Scores ranged from 1 (not meeting or addressing) to 3 (meeting/exceeding).

c Scores ranged from 1 (little to no extent) to 7 (to a great extent).

To assess innovativeness, effectiveness, and sustainability, 2 methods were systematically applied across all 19 interventions. Because the criteria for the innovativeness and effectiveness domains were based on several sources, a scoring matrix was created that included all 6 innovativeness criteria and 5 effectiveness criteria. To assess sustainability, a recently developed self-report tool for program sustainability was used (16,20).

To assess innovativeness and effectiveness, programs were rated 1 or 2 years into implementation. Using the scoring matrix with all 11 criteria, independent raters assessed how well individual programs addressed each criterion. Three independent raters familiar with the programs (MFH program officer, evaluation team member, and dissemination team member) (matrix available on request from the corresponding author) were trained to assess each program. The training defined and described the individual criteria and provided examples of each. Raters were instructed to rate the intervention design, intervention and evaluation activities, and intervention outcomes on a scale from 1 (not meeting or addressing this criterion) to 3 (meeting or exceeding this criterion). For example, interventions receiving a 2 in creating environmental or policy change (an innovativeness criterion) may have engaged in advocacy work or were planning for an environmental change but had not begun implementation. Similarly, a rating of 2 may indicate that the intervention indirectly addressed the criterion. For example, for addressing populations with health disparities (an innovativeness criterion), an intervention received a score of 3 if activities included special considerations for reaching that audience but received a score of 2 if this population was indirectly reached with intervention activities. Raters identified specific examples and data from each intervention to support their scores.

Once all interventions were rated on innovativeness and effectiveness, the 3 raters met and discussed each of their ratings. The purpose of this review was to gain consensus on the ratings by discussing each rater’s score, using examples and data from each intervention to support their scores. A score consensus was reached and was used as the final score for each criterion, ranging from 1 to 3.

To measure sustainability, the Program Sustainability Assessment Tool was electronically administered to measure whether a program had the necessary practices and processes to sustain itself (20). This tool was designed to help identify the strengths and weaknesses of an intervention’s sustainability efforts. Two to 3 respondents from each program self-reported on a scale of 1 (little to no extent) to 7 (to a great extent) the degree to which they felt their project met each criterion. An average of respondents from each program was used to create the sustainability score for each criterion.

Average scores for innovativeness, effectiveness, and sustainability were calculated (across criteria in each domain). The maximum score an intervention could achieve for innovativeness was 18 (6 criteria × 3 points), for effectiveness was 15 (5 criteria × 3 points), and for sustainability was 56 (8 criteria × 7 points) (Table 1). Innovativeness scores ranged from 7 to 16 (mean = 10.2), effectiveness scores ranged from 7 to 14 (mean = 10.2), and sustainability scores ranged from 22.3 to 52.5 (mean = 41.8). Domain-specific scores were then converted to percentages so that each domain contributed equally to the final score (Table 1). Each intervention received an average model practice building score by calculating the average percentage from each domain.

Five of the 19 interventions were selected for replication and dissemination and as “MFH model practices” because of their high average model practice building score relative to those of other grantees. We describe 2 interventions that were selected (Table 2).

**Table 2:** Sample Activities from Model Practice Programs

Type of Change	Example Activity
**Polk County Health Center**	
1. Healthy eating environment changes across 4 counties	Replaced soda with milk in 2 school vending machines
2. Physical activity environment changes across 5 counties	Developed a 3-mile multiuse trail with multiple connecting paths for trips of different lengths
3. School wellness policies updated	Passed joint use policies in 8 school districts to share facilities and equipment with community residents
**Trailnet**	
1. Capacity building and technical assistance	Conducted readiness and needs assessments in three neighborhoods, provided opportunities for professional development of Healthy, Active, and Vibrant Communities Initiative community leaders
2. Physical activity environment changes across 4 neighborhoods	Created plans for walking trails and implemented street improvements
3. Policies to support physical activity and healthy eating adopted	Adopted Complete Streets policies in 3 neighborhoods, enacted policies to allow farmers’ markets in one neighborhood

### Example 1: Polk County

In 2007, MFH funded the Polk County Health Center (PCHC), a community-based health and wellness service provider, to work in 18 rural communities in southwest Missouri counties. The Obesity Prevention Project promoted healthy eating and physical activity by implementing policy and environmental changes that support healthy behaviors in local schools and the greater community. Although the intervention targeted children and their families, environmental modifications targeted all community residents. The Obesity Prevention Project used the Missouri Department of Health and Senior Services’ Community Tobacco, Physical Activity, and Nutrition Policy and Environment Assessment and Resource Guide to identify appropriate environmental and policy recommendations (http://health.mo.gov/living/lpha/pdf/nuttobassresguide.pdf).

The objectives of the Obesity Prevention Project were to increase the number of opportunities for physical activity and healthy eating and assist communities in implementing policies to support these efforts. PCHC’s Obesity Prevention Project was selected as a model practice because it met several criteria of innovativeness, effectiveness, and sustainability. PCHC’s innovativeness score was 14 (78%), its effectiveness score was 9 (60%), and its sustainability score was 45.3 (81%) (Table 1). Its strengths in innovation were implementing environmental and policy strategies (eg, walking trails, worksite wellness policies) to increase physical activity and healthy eating in rural communities with health disparities and engaging 51 partners from 10 sectors (eg, schools, local businesses, community organizations) over 3 years (Table 2).

For effectiveness, PCHC used existing evidence from the literature recommending environmental and policy changes (21,22) and a community assessment to identify the environmental and policy strategies to be implemented in each of the 18 communities, and it increased the number of facilities that support physical activity or healthy eating (eg, exercise rooms, healthy vending machine options). Over 3 years, the number of opportunities or facilities for physical activity and healthy eating in the 18 communities increased by 19% (from 189 to 225 facilities).

Finally, PCHC reported having processes and procedures in place to sustain its efforts. Some of its strengths in sustainability included demonstration of funding stability, political support, community partnerships, and communication. PCHC secured more than $450,000 in additional funding for project activities and provided training opportunities on creating environmental and policy changes for 12 community leaders and 12 school superintendents to sustain support for physical activity and healthy eating in the community. PCHC was also successful in communicating project activities in various ways, including 17 published local newspaper articles on the implemented environmental changes in the targeted communities.

PCHC is replicating its obesity prevention efforts at worksites, a new setting. In collaboration with health department staff from several other counties, PCHC is working with the region’s employers to develop worksite wellness policies and improve existing policies that promote healthy eating and physical activity.

### Example 2: Trailnet’s HAVC Initiative

In 2008, MFH funded Trailnet’s Healthy, Active, and Vibrant Communities (HAVC) Initiative. Trailnet is a St. Louis–based nonprofit organization that promotes healthy and active living through walking and bicycling. Trailnet’s HAVC Initiative targeted residents of 3 low-income communities and focused on changing policy, the built environment, and social networks to create population-level change in physical activity and healthy eating.

One of Trailnet’s activities was the development of a tool kit that provides case studies, policy recommendations, and funding resources to help promote healthy and active living in communities. The tool kit has been recognized nationally by the Early Assessment of Programs and Policies to Prevent Childhood Obesity (Robert Wood Johnson Foundation) and the University of North Carolina’s Center TRT.

The objectives of the Trailnet HAVC Initiative were to

1. implement local action plans with task force leadership using the tool kit;
2. disseminate the Initiative’s model of working with communities to create institutional change;
3. develop a Bikeable-Walkable Community Master Plan as desired by each community;
4. host an annual walk-run event in each community; and
5. work with elementary schools in each community to improve school wellness.

The HAVC Initiative was selected as a model practice because it met several criteria of innovativeness, effectiveness, and sustainability. The program’s innovativeness score was 14 (78%), its effectiveness score was 11 (74%), and its sustainability score was 43.3 (77%) (Table 1).

Trailnet excelled in innovation by implementing several environmental and policy approaches and engaging more than 60 partners from diverse sectors (eg, government, nonprofit, schools, businesses). They worked with schools to adopt policies supporting physical activity, worked with local health departments to amend healthy eating policies, changed local policy to allow farmers markets in 1 neighborhood, adopted Complete Streets policies in 3 neighborhoods to design streets that accommodate all modes of transportation (eg, walking, cars), and developed Bikeable-Walkable Master Plans in each neighborhood (Table 2).

For effectiveness, the HAVC Initiative created community change by linking its activities with existing evidence. The HAVC Initiative was designed around the growing evidence base for physical activity promotion, using tools such as CDC’s *Guide to Community Preventive Services*, the Strategic Alliance’s ENACT (Environmental Nutrition and Activity Community Tool) tool, and best practices in community engagement (21–23). For sustainability, the HAVC Initiative presented and disseminated its program locally, regionally, and nationally. It leveraged additional funding for project activities, cultivated ownership in each of the 3 target communities, and developed organizational capacity to continue this work long-term.

The HAVC Initiative model continues to be implemented. Trailnet and its partners continue to host a yearly healthy living conference and have ongoing efforts to engage partners and community members. Trailnet received funding to work with an additional neighborhood on providing trail revitalization, educating local businesses about bicycle commuting, and developing a policy initiative to adopt land use and street design standards that accommodate pedestrians and bicyclists of all ages and abilities. Each of the 3 neighborhoods secured additional funding to sustain project activities.

## Discussion

As the rates of preventable chronic diseases continue to rise, public health foundations and practitioners need to shorten the time between conducting research and putting it into practice. They must be able to identify interventions for replication that have the potential to be effective without waiting for years of rigorous research and reviews of studies. The Institute of Medicine’s report *Progress in Preventing Childhood Obesity* calls for foundations to focus grants and funding opportunities on innovative projects that capitalize on cultural assets and demographic characteristics (19). The approach described operationalizes replication and dissemination criteria in response to the call from the Institute of Medicine and the desire of a local foundation to disseminate projects that will likely have an impact.

We operationalized criteria for identifying interventions into 3 domains (innovativeness, effectiveness, and sustainability) and rated interventions on all criteria within each domain. Five interventions were identified as model practices (only 2 are presented here), and MFH is actively disseminating these interventions. For example, Trailnet’s HAVC Initiative was used as the framework of another project undertaken by MFH, the Social Innovation Fund. This federally funded and locally matched project seeks to reduce the prevalence of obesity and tobacco use by addressing change at a community level using engagement strategies tested and tailored in the HAVC Initiative work. The approach described allowed MFH to identify a model practice intervention that could be replicated and used to leverage millions of dollars for the state.

Given the lack of practice-based evidence, the operationalization of replication and dissemination criteria described provides direction for continued behavior change efforts. Although the examples provided are for obesity-prevention programs, the criteria are broad enough to assess other community-based efforts (eg, substance abuse). Funders, practitioners, and researchers can apply these criteria to interventions to increase replication and dissemination rates.
